# Diabetes and COVID-19, a link revealed

**DOI:** 10.1093/lifemedi/lnac011

**Published:** 2022-06-28

**Authors:** Xiaoping Xiao, Liangqin Tong, Jonathan S Bogan, Penghua Wang, Gong Cheng

**Affiliations:** Section of Endocrinology, Department of Internal Medicine, and Department of Cell Biology, Yale School of Medicine, New Haven, CT 06520, USA; Ganzhou Key Laboratory for Drug Screening and Discovery, School of Geography and Environmental Engineering, Gannan Normal University, Ganzhou 341000, China; Department of Immunology, School of Medicine, the University of Connecticut Health Center, Farmington, CT 06030, USA; Institute of Infectious Diseases, Shenzhen Bay Laboratory, Shenzhen 518132, China; Ganzhou Key Laboratory for Drug Screening and Discovery, School of Geography and Environmental Engineering, Gannan Normal University, Ganzhou 341000, China; Tsinghua-Peking Joint Center for Life Sciences, School of Medicine, Tsinghua University, Beijing 100084, China

Infection with severe acute respiratory syndrome coronavirus-2 (SARS-CoV-2) presents highly heterogeneous clinical manifestations in humans [1]. Most infected individuals have asymptomatic, mild, or moderate disease. Nonetheless, elderly individuals and patients with diabetes mellitus and/or other comorbidities have a much higher risk of serious illness or even death after SARS-CoV-2 infection [2]. Diabetes mellitus is a metabolic disorder characterized by increased blood glucose concentrations. Numerous epidemiological surveys have shown that diabetes is a major risk factor for severe coronavirus disease 2019 (COVID-19). The rates of severity and mortality of COVID-19 were significantly higher in diabetic patients than in non-diabetic patients. While the underlying mechanism is complex, metabolic disturbances associated with diabetes could increase COVID-19 severity [2].

Serum metabolites play an important role in host metabolic and immune homeostasis. The dynamics of serum metabolites reflect host physiological and pathological states. Notably, numerous studies have shown that serum metabolites may regulate viral infection. Chenodeoxycholic acid (CDCA) is synthesized from cholesterol in the liver. Recently, CDCA has been shown to have an inhibitory activity against several viruses, including influenza A virus (IAV), rotavirus, and hepatitis B and D viruses [3]. CDCA suppresses the replication of multiple IAV subtypes by effectively restraining the nuclear export of viral ribonucleoprotein (vRNP) complexes. In addition, CDCA significantly reduces rotavirus infection by downregulating rotavirus-dependent lipid synthesis. Some bile salts, including CDCA, are capable of inhibiting sodium taurocholate co-transporting polypeptide (NTCP)-mediated hepatitis B virus (HBV) and hepatitis D virus (HDV) infection, since NTCP serves as a cellular receptor for the entry of these viruses. Both 25-hydroxycholesterol (25OHC) and 27-hydroxycholesterol (27OHC) are side-chain oxysterols that are physiologically present in human peripheral blood and have been shown to markedly suppress the replication of a large variety of both enveloped and non-enveloped human viruses.

Metabolomic studies with COVID-19 patients’ sera revealed that disturbed metabolic patterns were associated with the progression and severity of COVID-19 [4]. 1,5-Anhydro-D-glucitol (1,5-AG), a glucose-like pyran polyol, is an established serum marker for diabetes mellitus. The concentration of 1,5-AG in human serum is negatively correlated with the level of serum glucose, because glucose inhibits 1,5-AG reabsorption by the renal proximal tubule [5]. In addition to diabetic patients, serum 1,5-AG levels are lower in elderly people than in younger people [6]. However, the physiological consequences of reduced 1,5-AG concentration are unknown. In a recent study, Cheng and colleagues revealed a role of 1,5-AG in the control of SARS-CoV-2 infection [7]. The authors began by testing 222 commercially available human serum small-molecule metabolites one by one on SARS-CoV-2 infection. Seven compounds were identified with a potent antiviral activity, three of which were endogenous metabolites with little cytotoxicity. Notably, only 1,5-AG is associated with diabetes; therefore, the authors focused on characterizing it in depth. They demonstrated that 1,5-AG potently inhibited SARS-CoV-2 replication not only in several immortalized cell lines, but also in human lung epithelial organoids, a primary SARS-CoV-2 target tissue. Intriguingly, 1,5-AG is also present in the lung alveolar liquid, consistent with its physiological relevance to SARS-CoV-2 pathogenesis. Diabetes is associated with complex immunological and metabolic changes *in vivo* that could underlie the pathogenesis of severe COVID-19 complications. The authors then asked if 1,5-AG deficiency could be a major underlying mechanism. To this end, the authors employed a db/db mouse model derived from C57BL/KsJ mice, which are a mouse model of type 2 diabetes mellitus. Notably, the db/db mice were deficient in 1,5-AG and highly susceptible to SARS-CoV-2 infection. The db/db mice had much higher viral loads in their lung, trachea, and turbinate; showed perivascular, peribronchial, and alveolar inflammation, with massive infiltration of immune cells and alveolar damage, indicating severe pathological damage in the lung. Of note, 1,5-AG supplementation reduced severe illness and mortality caused by SARS-CoV-2 infection in db/db mice. The viral loads of db/db mice supplemented with 1,5-AG was reduced by approximately 100–1000 times, showing significantly less lung damage [7].

**Figure 1.**
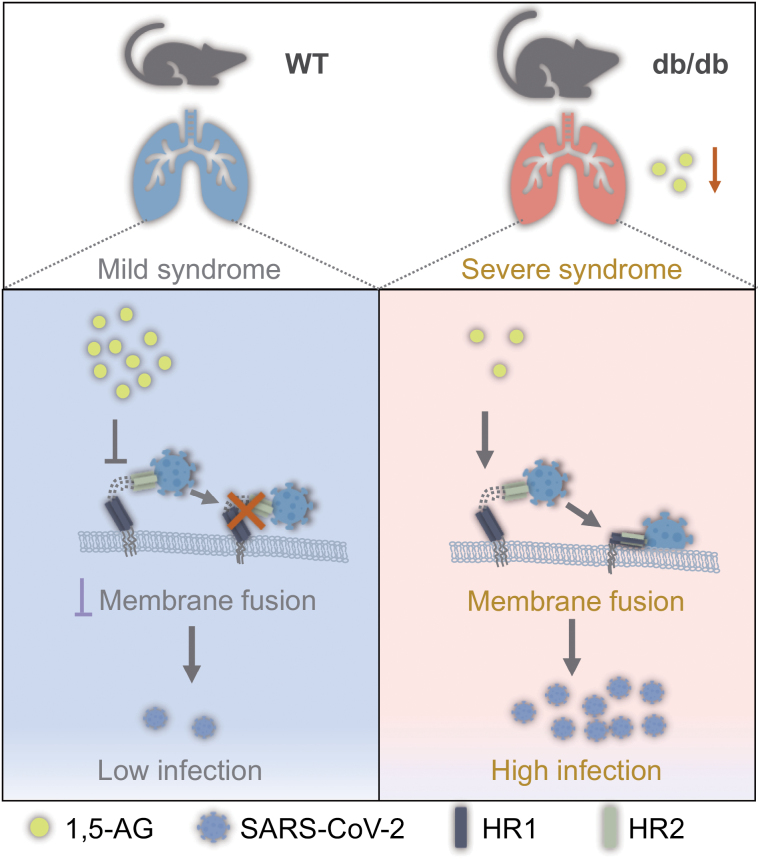
1,5-AG presents a potent anti-SARS-CoV-2 activity by interrupting membrane fusion. 1,5-AG deficiency in diabetic mice underlies severe COVID-19 pathogenesis.

The potential mechanism of anti-SARS-CoV-2 activity of 1,5-AG was further investigated. Previous studies have shown that elevated glucose levels and sustained aerobic glycolysis in human monocytes directly contribute to SARS-CoV-2 infection [8]; nonetheless, incubation with 1,5-AG did not affect glycolysis in Vero cells. Of note, 1,5-AG exerts its antiviral effect during viral entry. However, it does not interfere with SARS-CoV-2 attachment to host cells or directly block the interaction between the spike protein and human ACE2 (hACE2). Indeed, 1,5-AG significantly reduces viral internalization into host cells. According to bioinformatic docking modeling, 1,5-AG does not directly associate with the trimeric spike protein of SARS-CoV-2. Nonetheless, the model suggests that 1,5-AG may bind to the V952 and N955 sites in the HR1 domain of the S2 subunit. The heptad repeat 1 (HR1) and 2 (HR2) domains in the S2 subunit of the S protein bind to each other and form a six-helix bundle (6-HB) fusion core, which promotes viral–cellular membrane fusion. 1,5-AG presents a potent anti-SARS-CoV-2 activity by blocking the V952 and N955 sites of the heptad repeat 1 (HR1) domain of the SARS-CoV-2 spike protein, thereby interrupting 6-HB-mediated virus–host membrane fusion (Fig. 1). Of note, both V952 and N955 are highly conserved in all SARS-CoV-2 variants of concern (VOCs) and other coronaviruses, such as SARS-CoV and MERS-CoV, suggesting that 1,5-AG could be active against a broad-spectrum of coronaviruses. In addition to directly blocking coronaviral entry, 1,5-AG could decrease serum cholesterol levels, attenuate serum cytokine release, and protect db/db mice from lipopolysaccharide-induced pulmonary inflammation [9]. Thus, 1,5-AG may prevent the pathogenesis of severe COVID-19 in diabetic people by multiple mechanisms: blocking viral entry, maintaining cholesterol homeostasis, repressing inflammation.

Reduced serum 1,5-AG concentrations are not only a signature biomarker of diabetes but also are common in the patients with chronic renal failure, cirrhosis, and in the elderly [6], suggesting that this may be one factor to explain why these populations are also at high risk for severe COVID-19 disease. Considering the availability of 1,5-AG in many food sources [10], one promising approach may be to use 1,5-AG-containing food supplements in these vulnerable populations during the COVID-19 pandemic. Such an approach would require further study. Furthermore, maintenance of an effective *in vivo* level of 1,5-AG is challenging, as it is rapidly cleared in the urine in individuals with diabetes, since glucose is a strong competitor of 1,5-AG for reabsorption. It may be interesting to learn whether sodium-glucose cotransporter 2 inhibitors, which are commonly used to treat diabetes and inhibit renal glucose reabsorption, affect serum 1,5-AG concentrations. As well, future efforts may be directed to develop 1,5-AG analogs with long half-life and high bioavailability, as well as potent anti-SARS-CoV-2 activity. Finally, 1,5-AG might be administered together with insulin or other hypoglycemic drugs to sustain a high level of 1,5-AG in diabetic patients with COVID-19.
